# Genome-wide association study for response to vaccination in Angus calves^1^

**DOI:** 10.1186/s12863-018-0709-5

**Published:** 2019-01-08

**Authors:** L. M. Kramer, M. S. Mayes, E. D. Downey, R. G. Tait, A. Woolums, C. Chase, J. M. Reecy

**Affiliations:** 1 Department of Animal Science, Iowa State University, 2255 Kildee Hall, Ames, IA 50011 USA; 2 Elanco Animal Health, Larchwood, IA 51241 USA; 3 Neogen GeneSeek Operations, Lincoln, NE 68504 USA; 4 Department of Pathobiology and Population Medicine, Mississippi State University, Mississippi State, MS 39762 USA; 5 Department of Veterinary and Biomedical Sciences, South Dakota State University, Brookings, SD 57006 USA

**Keywords:** Accuracy, Beef cattle, Bovine respiratory disease complex, Genome-wide association study, Heritability, Immune response, Vaccination

## Abstract

**Background:**

Bovine respiratory disease complex (BRDC) is one of the most important sources of loss within the beef cattle industry in the USA. Steps have been taken to reduce the incidence of BRDC through vaccination. Despite the effectiveness of vaccines, large proportions of cattle still experience morbidity and mortality. Identification of genomic regions that are associated with variation in response to vaccination would allow for the selection of individuals genetically predisposed to respond to vaccination based on specific markers, while heritability and accuracy estimates would help facilitate genomic selection. This in turn may lead to selection for beef cattle herds that may have lower incidence rate of BRDC after vaccination. This study utilizes an Angus herd of more than 2000 head of cattle to identify these regions of association.

**Results:**

Genome wide association studies were performed for viral neutralization antibody level and response to vaccination traits against four different viruses associated with BRDC: bovine viral diarrhea virus 1 and 2 (BVDV1 and BVDV2), bovine respiratory syncytial virus (BRSV), and bovine herpesvirus (BHV1). A total of six 1-Mb windows were associated with greater than 1% of the genetic variance for the analyzed vaccination response traits. Heritabilities ranged from 0.08 to 0.21 and prediction accuracy ranged from 0.01 to 0.33 across 7 different vaccination traits.

**Conclusions:**

Although six 1-Mb windows were identified as associated with 1% or greater genetic variance for viral neutralization antibody level and response to vaccination traits, few genes around these windows could readily be considered candidates. This indicates the need for further functional genomic annotation, as these regions appear to be gene deserts. Traits ranged from lowly to moderately heritable, which indicated the potential for selection of individuals that are genetically pre-disposed to respond to vaccination. The relatively low amount of genetic variance accounted for by any 1-Mb window indicated that viral neutralization antibody level and response to vaccination traits are polygenic in nature. Selection for these traits is possible, but likely to be slow due to the low heritabilities and absence of markers with high genetic variation associated with them.

## Background

Bovine respiratory disease complex (BRDC) is one of the most costly and pervasive disease conditions facing beef cattle producers. With more than $750 million dollars in losses each year due to morbidity, mortality, and performance loss, vaccines have been one method used in an attempt to reduce overall incidence rates and by extension economic losses [1–4]. Despite widespread adoption of vaccination protocols in the beef cattle industry, BRDC remains extremely prevalent due to varying levels of vaccine efficacy [5, 6]. Research into response to vaccination has identified multiple environmental and management variables which impact an individual calf’s ability to mount an antibody response [7–9]. Factors such as maternally derived antibody levels from colostrum, calf age, seasonality, and weaning status all impact a calf’s individual immune system, and result in variability in its response to vaccination [10–12].

**Fig. 1:**
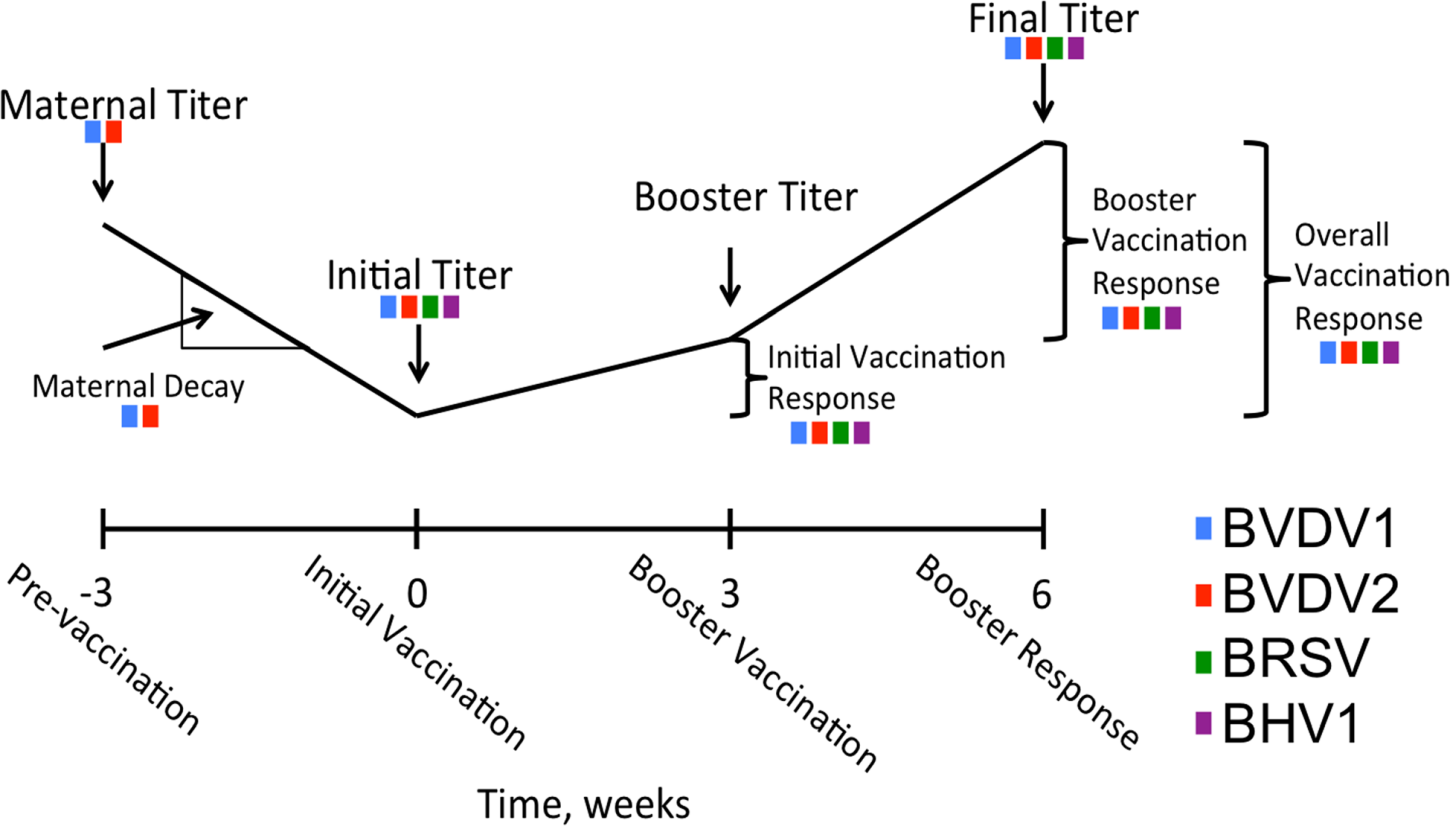
Study sample collection timeline. Colored boxes correspond to data availability of that trait to one of the four viruses. Bovine Viral Diarrhea Virus 1 (BVDV1) in blue, Bovine Viral Diarrhea Virus 2 (BVDV2) in red, Bovine Respiratory Syncytial Virus (BRSV) in green, Bovine Herpesvirus (BHV1) in purple. For each virus 3 response variables were calculated: 1) initial vaccination response = booster titer – initial titer; 2) booster vaccination response = final titer – booster titer; 3) overall vaccination response = final titer – initial titer. For BVDV1 and BVDV2 rate of maternal antibody decay was calculated: (initial titer – maternal titer) / number of days between initial titer and maternal titer measurements)

In order to improve efficacy of vaccination for BRDC, it is desired that all calves exhibit an adequate immune response after vaccination. If this is the case, calves would likely be better protected against future exposure to BRDC viruses after a multi-shot vaccination procedure and thereby result in less economic loss due to BRDC. One way to improve beef cattle response to vaccination may be through genomic selection. Previously, we reported on variables that influenced response to vaccination against bovine viral diarrhea virus 1 (BVDV1), bovine viral diarrhea virus 2 (BVDV2), bovine respiratory syncytial virus (BRSV), and bovine herpesvirus (BHV1) [13–15]. A genome-wide association study was performed on five different responses to vaccination/viral antibody titer traits across all four viruses (initial titer, final titer, initial vaccination response [IVR], booster vaccination response [BVR], overall vaccination response [OVR]) with an additional two traits analyzed only on BVDV1 and BVDV2 (maternal decay and maternal antibody titer) due to availability of data (Fig. 1). Through heritability and accuracy estimates for response to vaccination and viral antibody titer level traits, producers would be afforded the opportunity to select for cattle with higher immune response to vaccination. As such, the goal of this study was to identify regions of the genome that were associated with response to vaccination and viral antibody level and evaluate the potential for genomic selection for calves with improved immune response to vaccination as has been done in cattle [16] and other species [17].

## Results

### Estimates of genetic and residual variance, and heritability

Estimates of heritabilities and variances were obtained for each viral antibody titer levels and response to vaccination traits (Table 1). Heritability (h^2^) estimates, which were the proportion of additive genetic variance out of the total phenotypic variance, ranged from 0.08 to 0.22. The greatest spread of heritabilities was found within BVDV2 with a minimum estimated heritability of 0.08 and a maximum of 0.22. In contrast, BRSV had the smallest spread with a minimum heritability of 0.12 and a maximum of 0.18. Maternal decay and maternal antibody titer traits were only available for BVDV1 and BVDV2. Total genetic and residual posterior variances were relatively similar across all viral neutralization antibody level and response to vaccination traits. Maternal decay of BVDV2 titer level had the lowest posterior genetic variance (0.005) and residual variance (0.02). In contrast, no single antibody titer level or response to vaccination trait had both the highest genetic and residual posterior variance.

**Table 1:** Posterior estimates of genetic (σ_g_^2^) and residual (σ_e_^2^) variance, and heritability (h^2^)

Virus	Trait^a^	σ_g_^2^	σ_e_^2^	h^2^
BVDV1	Maternal Decay^b^	0.01	0.03	0.22
	Maternal Antibody Titer^b^	0.28	1.58	0.15
	Initial Titer	0.41	1.58	0.21
	Initial Vaccination Response	0.17	0.73	0.18
	Booster Vaccination Response	0.33	1.42	0.19
	Overall Vaccination Response	0.29	1.48	0.16
	Final Titer	0.33	1.65	0.17
BVDV2	Maternal Decay^b^	0.005	0.02	0.21
	Maternal Antibody Titer^b^	0.23	2.03	0.10
	Initial Titer	0.17	1.94	0.08
	Initial Vaccination Response	0.11	1.16	0.09
	Booster Vaccination Response	0.21	2.25	0.08
	Overall Vaccination Response	0.18	2.02	0.08
	Final Titer	0.20	2.01	0.09
BRSV	Initial Titer	0.18	1.38	0.12
	Initial Vaccination Response	0.07	0.35	0.16
	Booster Vaccination Response	0.09	0.42	0.18
	Overall Vaccination Response	0.09	0.47	0.16
	Final Titer	0.08	0.49	0.14
BHV1	Initial Titer	0.14	1.24	0.10
	Initial Vaccination Response	0.22	1.52	0.13
	Booster Vaccination Response	0.29	2.20	0.18
	Overall Vaccination Response	0.30	2.28	0.12
	Final Titer	0.27	2.36	0.10

^a^All trait titers measured in log_2_ transformations from serum neutralization dilutions. Maternal Decay measured as log_2_ titer change per day

^b^Maternal Decay and Maternal antibody titer data only available for BVDV1 and BVDV2 (Bayes C analysis)

### Whole genome association

**Fig. 2:**
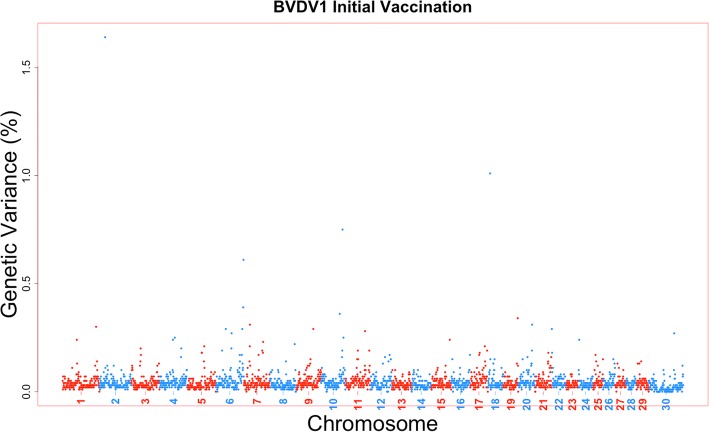
Manhattan plot for 1-Mb windows for Bovine viral diarrhea virus type 1 initial titer. A Manhattan plot showing every 1-Mb window by %variance accounted for by that window across the entire *Bos taurus* genome. Two singular windows exceeded the 1% genetic variance threshold: chromosome 2, Mb 24 with 1.64% genetic variance; chromosome 18, Mb 9 with 1.01% genetic variance

**Fig. 3:**
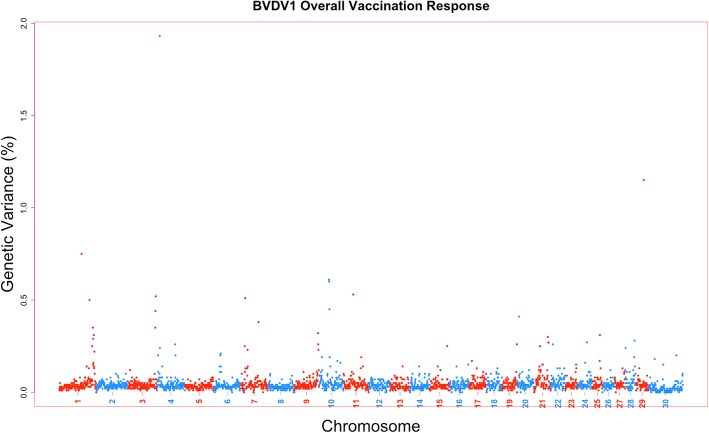
Manhattan plot for 1-Mb windows for Bovine viral diarrhea virus type 1 overall vaccination response. A Manhattan plot showing every 1-Mb window by %variance accounted for by that window across the entire *Bos taurus* genome. Two singular windows exceeded the 1% genetic variance threshold: chromosome 4, Mb 12 with 1.93% genetic variance; chromosome 29, Mb 32 with 1.15% genetic variance

**Fig. 4:**
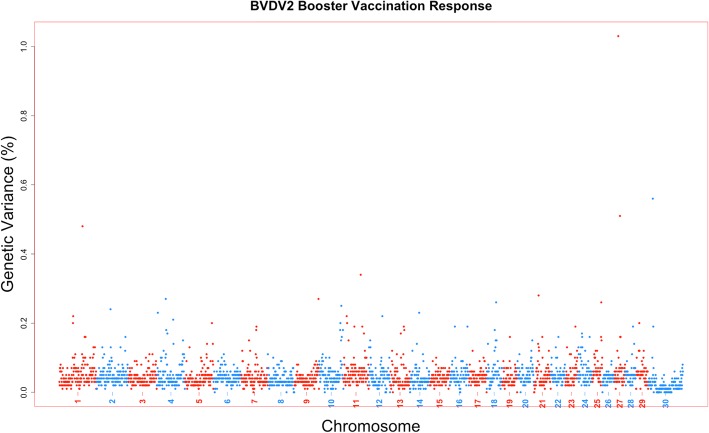
Manhattan plot for 1-Mb windows for Bovine viral diarrhea virus type 2 booster vaccination response. A Manhattan plot showing every 1-Mb window by %variance accounted for by that window across the entire *Bos taurus* genome. One window exceeded the 1% genetic variance threshold: chromosome 27, Mb 16 with 1.03% genetic variance

**Fig. 5:**
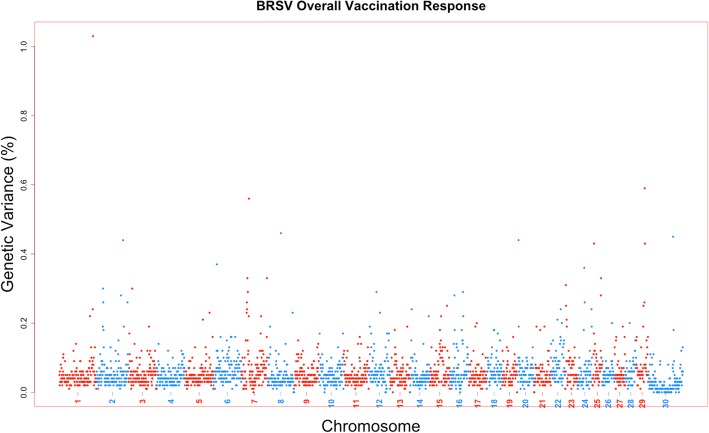
Manhattan plot for 1-Mb windows for Bovine respiratory syncytial virus overall vaccination response. A Manhattan plot showing every 1-Mb window by %variance accounted for by that window across the entire *Bos taurus* genome. One window exceeded the 1% genetic variance threshold: chromosome 1, Mb 144 with 1.03% genetic variance

The amount of genetic variance that every 1-Mb window across the bovine genome could account for was estimated based on the model for a viral neutralization antibody level or response to vaccination trait. Furthermore, the posterior probability of inclusion for each SNP and 1-Mb window was calculated as well. There were no 1-Mb regions across all viral neutralization antibody level and response to vaccination traits for all four viruses that exceeded a 0.9 posterior probability of inclusion. Six 1-Mb windows across the genome across traits could account for greater than 1% of the trait genetic variance (Table 2). The percent variance accounted by these windows ranged from 1.01 to 1.93%. These six 1-Mb windows were associated with four antibody titer levels or response to vaccination traits: two windows for BVDV1 initial titer (Fig. 2), two for BVDV1 overall vaccination response (Fig. 3), one for BVDV2 booster vaccination response (Fig. 4), and the final 1-Mb window for BRSV overall vaccination response (Fig. 5).

**Table 2:** 1-Mb genomic windows that accounted for greater than 1% genetic variance for response to vaccination traits

Virus	Trait^a^	Chr_Mb^b^	Starting SNP	Ending SNP	# of SNP	Genetic Variance (%)	PPI^c^
BVDV1	Initial Vaccination Titer	2_24	rs137131604	rs109222292	220	1.64	0.57
		18_9	rs43715906	rs29024678	400	1.01	0.65
	Overall Vaccination Response	4_12	rs133210106	rs109951163	172	1.93	0.61
		29_32	rs43727482	rs137609870	253	1.15	0.51
BVDV2	Booster Vaccination Response	27_16	rs134611614	rs43207573	300	1.03	0.54
BRSV	Overall Vaccination Response	1_144	rs109534947	rs42965155	296	1.03	0.49

^a^All trait titers measured in log_2_ transformations from serum neutralization dilutions

^b^1-Mb window defined by chromosome and mega base position. UMD3.1 build of *Bos taurus* genome

^c^Posterior probability of inclusion (inclusion rate in BayesB analysis)

All genes within these windows were evaluated as potential candidate genes. Unfortunately, none of the genes within these windows were annotated with immune function Gene Ontology terms. However, higher order gene families names, e.g. TRIML, may have some function related to immune system response [18]. Additionally, multiple transcription factors were identified such as EST1, which may have some functional relationship with immune response.

Traits were also analyzed categorically, with phenotypes being set as their integer value. This marginally improved the heritability of the response to vaccination and viral neutralization antibody levels (Table 3). However, in these analyses no windows accounted for greater than 1% of the estimated genetic variation.

**Table 3:** Categorical analysis posterior estimates of genetic (σ_g_^2^) and residual (σ_e_^2^) variance, and heritability (h^2^)

Virus	Trait^a^	σ_g_^2^	σ_e_^2^	h^2^
BVDV1	Maternal Antibody Titer^b^	0.33	1.00	0.25
	Initial Vaccination	0.22	1.00	0.18
	Initial Vaccination Response	0.22	1.00	0.18
	Booster Vaccination Response	0.15	1.00	0.13
	Overall Vaccination Response	0.16	1.00	0.14
	Final Titer	0.52	1.00	0.34
BVDV2	Maternal Antibody Titer^b^	0.24	1.00	0.19
	Initial Vaccination	0.15	1.00	0.13
	Initial Vaccination Response	0.11	1.00	0.10
	Booster Vaccination Response	0.11	1.00	0.10
	Overall Vaccination Response	0.11	1.00	0.10
	Final Titer	0.27	1.00	0.21
BRSV	Initial Vaccination	0.61	1.00	0.38
	Initial Vaccination Response	0.17	1.00	0.14
	Booster Vaccination Response	0.51	1.00	0.34
	Overall Vaccination Response	0.29	1.00	0.23
	Final Titer	0.47	1.00	0.32
BHV1	Initial Vaccination	0.61	1.00	0.38
	Initial Vaccination Response	0.14	1.00	0.12
	Booster Vaccination Response	0.14	1.00	0.13
	Overall Vaccination Response	0.15	1.00	0.13
	Final Titer	0.51	1.00	0.34

^a^All trait titers measured in log_2_ transformations from serum neutralization dilutions

^b^Maternal antibody titer data only available for BVDV1 and BVDV2

### Correlations and accuracies

Individuals were separated into five groups that exhibited high genetic diversity between groups while maintaining genetic similarity within groups. The number of individuals in each of the five groups can be seen in Table 4. Different numbers of individuals were present in the different clusters between different viral antibody titer levels and responses to vaccination traits was due to different numbers of calves with phenotype data and the presence or absence of various data time points. BVDV2 was the initial viral antigen studied at the inception of this study. As such, the individuals that could be analyzed for the other 3 viral antigens came from a smaller pool and are therefore more variable in the total number utilized. The pooled correlation between direct genetic value estimates and the true phenotype ranged from − 0.10 for BHV1 overall vaccination response to 0.09 for BVDV1 final titer and overall vaccination response. Prediction accuracies ranged from an absolute value of 0.002 to 0.31 (Table 5). Maternal decay traits for BVDV1 and BVDV2 were unable to be estimated due to a failure to converge, and so could not be calculated for correlation or accuracy.

**Table 4:** Number of individuals for each K Means group within response to vaccination trait

		K Means Groups					Total
Virus	Trait^a^	1	2	3	4	5	
BVDV1	Maternal Decay^b^	131	89	126	90	133	569
	Maternal Antibody Titer^b^	130	88	125	90	133	566
	Initial Vaccination	314	289	306	290	319	1518
	Initial Vaccination Response	244	236	253	234	273	1240
	Booster Vaccination Response	211	214	213	208	245	1091
	Overall Vaccination Response	198	187	210	206	243	1044
	Final Titer	234	236	238	240	281	1229
BVDV2	Maternal Decay^b^	198	194	150	161	192	895
	Maternal Antibody Titer^b^	232	210	185	195	227	1049
	Initial Vaccination	408	353	291	354	332	1738
	Initial Vaccination Response	426	353	314	371	351	1815
	Booster Vaccination Response	425	350	313	371	347	1806
	Overall Vaccination Response	424	350	312	372	347	1805
	Final Titer	425	350	312	372	347	1805
BRSV	Initial Vaccination	312	285	312	281	318	1508
	Initial Vaccination Response	268	217	248	230	246	1209
	Booster Vaccination Response	250	200	230	214	222	1116
	Overall Vaccination Response	286	256	273	252	270	1337
	Final Titer	287	263	276	261	280	1367
BHV1	Initial Vaccination	312	283	301	280	315	1491
	Initial Vaccination Response	312	285	302	281	318	1498
	Booster Vaccination Response	254	230	243	218	234	1179
	Overall Vaccination Response	256	236	253	226	245	1216
	Final Titer	257	244	256	234	254	1245

^a^All trait titers measured in log_2_ transformations from serum neutralization dilutions. Maternal Decay measured as log_2_ titer change per day

^b^Maternal Decay and Maternal antibody titer data only available for BVDV1 and BVDV2

**Table 5:** Pooled correlation between phenotype and predicted direct genetic value, and accuracy of prediction

Virus	Trait^a^	Correlation	Accuracy^b^
BVDV1	Maternal Antibody Titer^c^	0.02	0.04
	Initial Vaccination	0.06	0.12
	Initial Vaccination Response	0.02	0.05
	Booster Vaccination Response	0.08	0.19
	Overall Vaccination Response	0.09	0.22
	Final Titer	0.09	0.22
BVDV2	Maternal Antibody Titer^c^	− 0.05	0.15
	Initial Vaccination	−0.02	0.08
	Initial Vaccination Response	0.04	0.15
	Booster Vaccination Response	−0.01	0.03
	Overall Vaccination Response	0.00	0.00
	Final Titer	−0.03	0.11
BRSV	Initial Vaccination	0.07	0.22
	Initial Vaccination Response	0.00	0.01
	Booster Vaccination Response	−0.05	0.11
	Overall Vaccination Response	0.00	0.01
	Final Titer	0.01	0.04
BHV1	Initial Vaccination	0.02	0.05
	Initial Vaccination Response	0.05	0.13
	Booster Vaccination Response	0.01	0.03
	Overall Vaccination Response	−0.10	0.31
	Final Titer	− 0.06	0.19

^a^All trait titers measured in log_2_ transformations from serum neutralization dilutions

^b^Accuracy calculated as correlation between direct genetic value and phenotype divided by square root of heritability

^c^Maternal antibody titer data only available for BVDV1 and BVDV2

## Discussion

In this study, we set out to identify regions of the genome that were associated with viral neutralization antibody level and response to vaccination traits for four viruses associated with BRDC. To do so, a genome wide association study was performed to characterize regions based on the proportion of genetic variance they could account for within a trait. Heritability estimates for viral neutralization antibody level and response to vaccination traits were lowly to moderately heritable. Ranging from 0.08 to 0.22, we determined that the heritability of viral neutralization antibody level and response to vaccination traits varied the most in BVDV1 and BVDV2, and the least in BRSV. However, when only traits measured on all four viruses are compared, BVDV1 and BVDV2 have a much more concentrated range in heritabilities than the other two. Additionally, as described in Kramer et al. 2017, BRSV was the singular virus out of the four studied that did not contain individual calves that failed to seroconvert, potentially due to the ubiquitous nature of BRSV [16, 19]. This represented a smaller range in the titer sample data and may impact its ability to be compared to the other three viruses. Heritability estimates for immune related traits in other studies range from lowly (0.11) to highly heritable (0.47), dependent on the immune trait evaluated, whether it is due to a infectious challenge or vaccination, and species [20–24]. The heritability estimates based on estimated direct genetic variation are on the lower end of this previously found range. This is likely due to response to vaccination as a complex trait with many non-genetic influences as noted in Kramer et al. 2017, and will make selection for these antibody titer level and response to vaccination traits slower than if they were more heritable.

Low correlations were calculated between direct genetic value and the true phenotype, with a range of − 0.10 to 0.09. Accuracies also ranged from an absolute value of 0.002 to 0.31. These accuracies are a direct result of low heritabilities as accuracy was calculated as correlation divided by the square root of heritability. While the correlation between true and predicted phenotype is low, and accuracies are on average 0.11, some traits such as BHV1 overall vaccination response and BVDV1 final titer may have a level of accuracy such that they could be utilized in a selection index. These determined correlations and accuracies underscore the point that responses to vaccination traits remain complicated even after fitting management practices and other environmental and physiological factors in a model. With a greater number of individuals, it may be possible to improve the accuracy closer to its maximum, although it will be capped by the heritability estimates of response to vaccination traits (0.28–0.45 range for maximums). If accuracies of genomic predictions can be increased towards these maximums, then the rate of genetic progress for response to vaccination and viral neutralization antibody level traits will increase.

These traits appear to be highly polygenic, as no singular region was able to account for a large proportion of genetic variation and only six regions were estimated to account for more than 1% of that variation (Figs. 2, 3, 4, 5). The polygenic nature of viral neutralization antibody level, response to vaccination, and immune related traits is something that has been previously reported in numerous studies such as those focused on porcine [17, 25], chicken [26, 27], cattle [28–31], and mice [32]. In the six windows that accounted for more than 1% of the genetic variance in this study, no candidate genes were identified as none of the genes in those regions were annotated with Gene Ontology terms associated with immune response functions. The TRIM gene family is present, however, and may have some further functional relevance that is not currently described in published literature [18].

## Conclusions

This study aimed to examine response to vaccination against viruses associated with BRDC in Angus calves using genome wide association studies. Vaccination is one of the most widely used tools by producers to reduce and/or prevent BRDC incidence in herds. Efficacy remains lower than desired; therefore selection of cattle for response to vaccination could increase the effectiveness of vaccine use. This would potentially reduce losses associated with BRDC and improve animal welfare. Using serum samples across a 6–9 week period we were able to identify genomic regions associated with multiple response to vaccination traits, and determine the predictive accuracy of response to vaccination. Windows of size 1-Mb were found to account for up to 1.93% of genetic variation, indicating the polygenic nature of response to vaccination. With low to moderate heritabilities across response to vaccination traits, there appears to be room for selection of animals with improved vaccination response. Genes within the associated regions had little functional annotation associated with them, which may be resolved in future builds of the *Bos taurus* genome along with the help of the Functional Annotation of Animal Genomes (FAANG) initiative. All of this together provides a solid foundation for genetic variation within response to vaccination in Angus calves, and a basis for selection procedures.

## Methods

### Population

Calves (*n* = 2518) from a purebred American Angus herd located at Iowa State University Ames, IA, were used in this study. Not all individuals were utilized for all response to vaccination traits due to limited availability of recorded data at specific time points and non-genotyped calves. Therefore, the number of calves analyzed for each response to vaccination trait was less than the total number of genotyped calves. Calves were born in either a spring season or fall season classification, and across multiple years (2006–2012, 2014).

### Phenotypic data

Phenotypic data was collected as described in previous work [13–15]. Briefly; individuals were vaccinated with a modified live vaccine (Bovi-Shield Gold 5, Zoetis, Inc. Parsippany NJ). Serum samples were collected from the calves at multiple time points and then a viral neutralization assay was performed for each sample to quantify the level of antibodies present against one of four viruses (BVDV1, BVDV2, BRSV, BHV1). Dilutions were performed to identify the greatest dilution where neutralizing antibodies could still be detected, and a log_2_ titer was recorded. This was based on the Spearman-Kärber method for initial calculation [33]. Response traits were calculated as the change in titer values between serum sample collection time points (initial, booster, and overall vaccination response), or as the calculated titer value for a given time (maternal, initial, and final antibody titer).

### Genotypic data

The BovineSNP50 BeadChip (Illumina, San Diego, CA) and BovineHD BeadChip (Illumina, San Diego, CA) were used by Neogen GeneSeek Operations (Lincoln, NE) to perform SNP genotyping. Individuals were imputed by using the SNPipeline package (Hailin Su, https://github.com/cbkmephisto/SNPipeline) and FImpute [34] from the BovineSNP50 BeadChip to the BovineHD BeadChip using 820 Angus individuals genotyped natively for the Bovine HD BeadChip, both within and outside the ISU herd. Accuracy of imputation was about 97%, and was tested by randomly removing 5000 markers from the 50 k genotype, and then comparing imputed genotypes of these 5000 markers to the original genotypes. 574,662 single nucleotide polymorphisms (SNPs) remained after editing for a minor allele frequency (MAF) of 0.05. All SNPs were assigned a UMD3.1 bovine genome build position [35].

### Statistical model

All 574,662 SNPs were used alongside phenotypic titer measurements or differences to determine an estimate of each SNP effect for a respective trait. Single SNP effect estimates were obtained using BayesB approach through the program GenSel [36]. This method assumes a model in which a portion of SNP markers have zero effect on a trait, denoted as a π, and markers not assigned zero effect are used in prediction by estimating individual SNP effects. Models were generally fit as follows:

$$ y= Xb+ Zu+e $$

where y was a vector of response to vaccination trait observations; b is a set of vectors of fixed effects; u is a vector of SNPS as random effects sampled through BayesB; and e was the remaining vector of residuals corresponding to each phenotypic record [37]. Fixed effects and Covariates are explained in detail within, but included the fixed effects of: Year-Season classification, Sex, Dam Age, Weaned Status, Pink Eye Status; covariates of Calf Age within Year-Season classification, Titer score, Titer-by-Titer score, and Average Daily Gain [13]. A π value of 0.999 was used for BayesB [38] analysis of each trait (approximately 575 SNP markers with a non-zero effect), while genetic and residual variances for each trait were estimated using BayesC (initial variances set as half the total phenotypic variance) before being used in BayesB [37, 39]. A total of 50,000 iterations were used with Monte Carlo Markov Chain, with the first 5000 thrown out as a burn in, to obtain posterior means of SNP marker effects and posterior probabilities of inclusion.

BayesB analysis outputs included SNP marker effect estimates, 1-Mb window effect estimates, and a direct genetic value prediction for every individual. Each SNP marker and 1-Mb window was estimated a % genetic variance that it accounted for, and a posterior probability of inclusion to indicate the frequency in which it was included in the BayesB analysis.

Response to vaccination and viral titer level traits were also analyzed as categorical traits in addition to the above analysis, and models for these traits as categorical were fit and run as above. Phenotypes were set as the truncated integer value for a recorded phenotypic measurement (1.0–1.99 = 1, etc.…) rather than rounding to integer values. All other fixed effects, covariates, and the matrix of random marker effects remained the same.

### Heritability

Heritability estimates for each trait were obtained through BayesC with a π value of 0. This was chosen due to BayesB shrinking small effects to allow for the detection of relatively larger effects, and therefore biasing heritability estimates [37, 39]. Heritability was calculated as the posterior genetic variance over the total estimated posterior phenotypic variance.

### Accuracy of prediction

Prediction accuracies were estimated through cross validation for the direct genetic values that were predicted for each individual in BayesC. Genotyped individuals were initially classified into 9 unequal groups through K-means clustering [40] after creating a genomic relationship matrix [41]. Using this method, each group was made to be as genetically similar as possible within group, while remaining genetically distinct to the other 8 groups. Through this, groups can be used as predictors of other groups due to genetic similarity. The 9 initial groups were clustered down to 5 total groups by merging numerically smaller groups together so that groups were more consistently sized. BayesC with a π value of 0 was used to perform training and validation for these groups as follows: 1 group was excluded from the BayesC π = 0 analysis, and direct genetic values were calculated on the remaining 4 groups of animals. Those single SNP marker estimates were then used validate the final 5th group. Each group was rotated so that every individual was validated once and used as training set four times. Accuracy was calculated by taking the correlation between predicted direct genetic value and phenotypes for all individuals in a given trait divided by the square root of heritability for that trait.

### Candidate gene identification

The 1-Mb genomic regions accounting for more than 1% OF genetic variation for response to vaccination traits were identified for candidate gene investigation. A region extending half a mega base on either side of the identified 1-Mb regions, for a total of 2 Mb in length, was analyzed by looking at the biological function of every gene currently annotated in the UMD 3.1 *Bos taurus* assembly of the genome.

## Abbreviations

BHV1: Bovine Herpesvirus
BRSV: Bovine Respiratory Syncytial Virus
BVDV1: Bovine Viral Diarrhea Virus Type 1
BVDV2: Bovine Viral Diarrhea Virus Type 2
BVR: Booster Vaccination Response
GWAS: Genome-wide Association Study
IVR: Initial Vaccination Response
MAF: Minor Allele Frequency
OVR: Overall Vaccination Response
SNP: Single Nucleotide Polymorphism
